# 

**Published:** 1905-07-08

**Authors:** 


### NOTICE TO CORRESPONDENTS.

All MSS., Letters, Books for Review, and other matters intended for the Editor should be addressed The Editor, "The Hospital," The Hospital Buildings, 28 & 29 Southampton Street, Strand, W.C.

The Editor cannot undertake to return rejected MS., even when accompanied by stamped directed envelope.

**Notice to Advertisers and Subscribers.**

All Advertisements, Orders for Copies of The Hospital, and Business Communications should be addressed to **THE MANAGER (Not to the Editor),** The Hospital, 28 & 29 Southampton Street, Strand, London, W.C.

The Cost of Subscription, post free, is at follows:—Per week, 3½d.; per uarter, 3s. 3d.; per half-year, 6s. 6d.; per annum, 13s. Foreign and olonial:—Per annum, 19s. 6d., payable, in all cases, in advance.

**NOTICE.—Vol. XXXVII. of "The Hospital" October 1904 to March 1905), in handsome cloth binding, will shortly be on sale at the office of this Journal, price 10s. 6d. Cases for binding the Numbers contained in this Volume 2s. Index to Vol. XXXVII., 3½d., post free.**

**The Monthly Part for July is now ready. Price 1s., by post, 1s. 3d.**

### Standard Nursing Manuals.

**A HANDBOOK FOR NURSES.**
By J. K. Watson, M.D., M.B., C.M.
Second Edition. Crown 8vo. over 400 pp. profusely illustrated, **5s.** net, **5s. 5d.** post free.

**ELEMENTARY ANATOMY AND SURGERY FOR NURSES.**
By William McAdam Eccles, M.S.Lond., M.B., &c.
Crown 8vo. Illustrated, cloth, **2s. 6d.** post free.

**A COMPLETE HANDBOOK OF MIDWIFERY.**
By J. K. Watson, M.D., M.B., C.M.
Crown 8vo. over 300 pp. profusely illustrated with 143 Diagrams. **6s.** net, **6s. 4d.** post free.

**ELEMENTARY PHYSIOLOGY FOR NURSES.**

By C. F. Marshall, M.D., B.Sc., F.R.C.S.

Crown 8vo. Illustrated, cloth, **2s.** post free.

**NURSING: ITS THEORY AND PRACTICE.**
By Percy G. Lewis, M.D., M.R.C.S., L.S.A.
New and Enlarged Edition.
Crown 8vo. over 400 pp. profusely illustrated, **3s. 6d.** post free.

**MIDWIVES' POCKET BOOK.**
(With Appendix containing particulars and rules of the Central Midwives Board.)
By Honnor Morten.
Small crown 8vo. cloth, **1s.** post free.

**OPHTHALMIC NURSING.**
By Sydney Stephenson, M.B., F.R.C.S.E.
Second Edition. Crown 8vo. illustrated, with glossary of terms, cloth, **3s. 6d.** net, **3s. 10d.** post free.

**HOSPITAL SISTERS AND THEIR DUTIES.**
By Eva C. E. Lückes.
Third Edition, Enlarged and partly Rewritten. Crown 8vo. 200 pp. cloth gilt, **2s. 6d.** post free.

**NURSING IN DISEASES OF THE THROAT, NOSE AND EAR.**

By P. Macleod Yearsley, F.R.C.S.Eng., M.R.C.S., L.R.C.P.Lond.

Demy 8vo. Illustrated, cloth, **2s. 6d.** post free.

**SURGICAL WARD-WORK AND NURSING.**
By Alexander Miles, M.D.Edin., C.M., F.R.C.S.E.
Revised Edition. Demy 8vo. Illustrated, cloth gilt, **3s. 6d.** net, **3s. 10d.** post free.

**A PRACTICAL HANDBOOK OF MIDWIFERY.**
By Francis W. Haultain, M.D.
New and Revised Edition. Illustrated. Handsomely bound in leather, **6s.** net, **6s. 3d.** post free.

**PRACTICAL GUIDE TO SURGICAL BANDAGING AND DRESSINGS.**
By Wm. Johnson Smith, F.R.C.S.
Demy 16mo., profusely Illustrated (suitable for apron pocket), cloth, **2s.** post free.

*COMPLETE CATALOGUE POST FREE ON APPLICATION.*

The SCIENTIFIC PRESS, LTD., 28 & 29 Southampton Street, Strand, London, W.C.

### The NURSING MIRROR.

**The Nurse's Own Paper.**

**Price ONE PENNY Weekly.**

Of all Booksellers and Newsagents, or posted direct from the Office.

*Write for Subscription Rates to The Manager,*

**THE NURSING MIRROR, The Hospital Building, 28 & 29 Southampton Street, Strand, London, W.C.**

